# DeepAutoGlioma: a deep learning autoencoder-based multi-omics data integration and classification tools for glioma subtyping

**DOI:** 10.1186/s13040-023-00349-7

**Published:** 2023-11-15

**Authors:** Sana Munquad, Asim Bikas Das

**Affiliations:** Department of Biotechnology, National Institute of Technology Warangal, Warangal, Telangana 506004 India

**Keywords:** Multi-omics, Autoencoder, Lower-grade glioma (LGG), Glioblastoma multiforme (GBM), Convolutional neural network (CNN)

## Abstract

**Background and objective:**

The classification of glioma subtypes is essential for precision therapy. Due to the heterogeneity of gliomas, the subtype-specific molecular pattern can be captured by integrating and analyzing high-throughput omics data from different genomic layers. The development of a deep-learning framework enables the integration of multi-omics data to classify the glioma subtypes to support the clinical diagnosis.

**Results:**

Transcriptome and methylome data of glioma patients were preprocessed, and differentially expressed features from both datasets were identified. Subsequently, a Cox regression analysis determined genes and CpGs associated with survival. Gene set enrichment analysis was carried out to examine the biological significance of the features. Further, we identified CpG and gene pairs by mapping them in the promoter region of corresponding genes. The methylation and gene expression levels of these CpGs and genes were embedded in a lower-dimensional space with an autoencoder. Next, ANN and CNN were used to classify subtypes using the latent features from embedding space. CNN performs better than ANN for subtyping lower-grade gliomas (LGG) and glioblastoma multiforme (GBM). The subtyping accuracy of CNN was 98.03% (± 0.06) and 94.07% (± 0.01) in LGG and GBM, respectively. The precision of the models was 97.67% in LGG and 90.40% in GBM. The model sensitivity was 96.96% in LGG and 91.18% in GBM. Additionally, we observed the superior performance of CNN with external datasets. The genes and CpGs pairs used to develop the model showed better performance than the random CpGs-gene pairs, preprocessed data, and single omics data.

**Conclusions:**

The current study showed that a novel feature selection and data integration strategy led to the development of DeepAutoGlioma, an effective framework for diagnosing glioma subtypes.

**Supplementary Information:**

The online version contains supplementary material available at 10.1186/s13040-023-00349-7.

## Introduction

The accurate classification of brain tumors is crucial in modern clinical practice. For clinical decision-making, it is vital to distinguish various types of brain tumors, such as subtypes and primary gliomas, from metastases. Presently, the characterization of brain tumors is mainly performed based on imaging and histopathology [1–3]. Due to the heterogeneity of neoplastic brain tissue and the mixed characteristics of gliomas, it is often difficult to separate the subtype by imaging. Furthermore, most histopathology-based procedures suffer from intraobserver and interobserver variability, resulting in suboptimal clinical outcomes [4, 5]. As a result, new clinical variables are required for the stratification of gliomas for precision therapy. Gliomas are the most prevalent type of brain tumor, accounting for approximately 33% of all cases. Gliomas are classified according to how rapidly or slowly the cells divide. Slower-growing gliomas are known as lower-grade gliomas (LGG), whereas more aggressive gliomas are named glioblastoma multiforme (GBM), a Grade IV cancer. LGG is more common in younger people; instead, GBM is more frequently diagnosed in older patients. The LGG is a grade II and III tumor classified into three subtypes: astrocytoma, oligodendroglioma, and oligoastrocytoma. Some of these LGGs develop into GBM, while others remain in the same stage for an extended period [6, 7]. Similarly, there are three subtypes of GBM, i.e., classical, proneural, and mesenchymal [8]. Due to the diverse subtypes of lower and higher-grade gliomas, an effective stratification methodology is required for improved diagnosis. Perturbations at various molecular layers (such as gene expression, methylation, etc.) result in the emergence of all types of human cancer. Moreover, in gliomas, the quantum of molecular heterogeneity is too high, posing a further challenge to early detection and understanding of disease etiology. In this aspect, analysis of high-throughput omics data from different molecular layers can decipher the link between molecular signatures and cancer phenotypes. Indeed, multi-omics data integration can elucidate how the molecular alterations at different layers contribute to disease formation and provide a global view of the molecular signature of disease. Previous studies have primarily relied on mono-omics data, specifically gene expression data, to develop a model for classifying glioma subtypes. Consequently, these models fail to account for other molecular changes in glioma, such as epigenetic modifications, which play a significant role in the development of cancer [9–11]. The epigenetic modifications (or methylations) directly regulate the transcriptomic landscape of the cell. Hypermethylation of CpG sites on promoter regions generally reduces gene expression, whereas hypomethylation elevates gene expression [12, 13]. Therefore, the biologically relevant diagnostic model can be developed by integrating these two interlinked biological phenomena. The integration of multi-omics data is a great challenge, and powerful integration methods can provide an efficient diagnostic tool to support the clinician [14]. Recently, deep learning (DL) models have been successfully applied to integrate high-dimensional genomics and epigenomics data. When analyzing such data, a common approach is to find data embedded in a lower-dimensional space. The compressed features from embedding space can be good predictors in predictive models. Autoencoder is a deep learning-based nonlinear embedding approach recently implemented to integrate multi-omics data to develop diagnostic and predictive models [15–17]. In the present study, we developed an autoencoder and deep-neural network-based, biologically relevant novel approach for integrating the transcriptome and methylome and subsequently classified the subtypes of LGG and GBM. To this end, we first identified the differentially expressed genes (DEGs) and differentially methylated regions (DMRs). Then we performed the univariate Cox regression analysis to determine the DEGs and DMRs associated with patient survival. Next, we mapped the DMR to the DEG through the promoter regions to find the altered methylation affecting gene expression. Finally, we implemented an autoencoder with concatenated inputs to integrate the survival-linked genes and CpGs into promoters. The autoencoder reduces the dimensionality of the corresponding gene expression and methylation matrices. Then, the new feature from the autoencoder was used to make DL-based models for subtyping. The current framework achieves a superior accuracy of > 94% for subtyping LGG and GBM. The framework is called DeepAutoGlioma. The present work introduces a new way for subtyping brain cancer, and we think this research will shed light on the DL-based clinical support system for accurate disease prediction using multi-omic data.

## Results

### Identification of biologically relevant features for classification of LGG and GBM subtypes

**Fig. 1:**
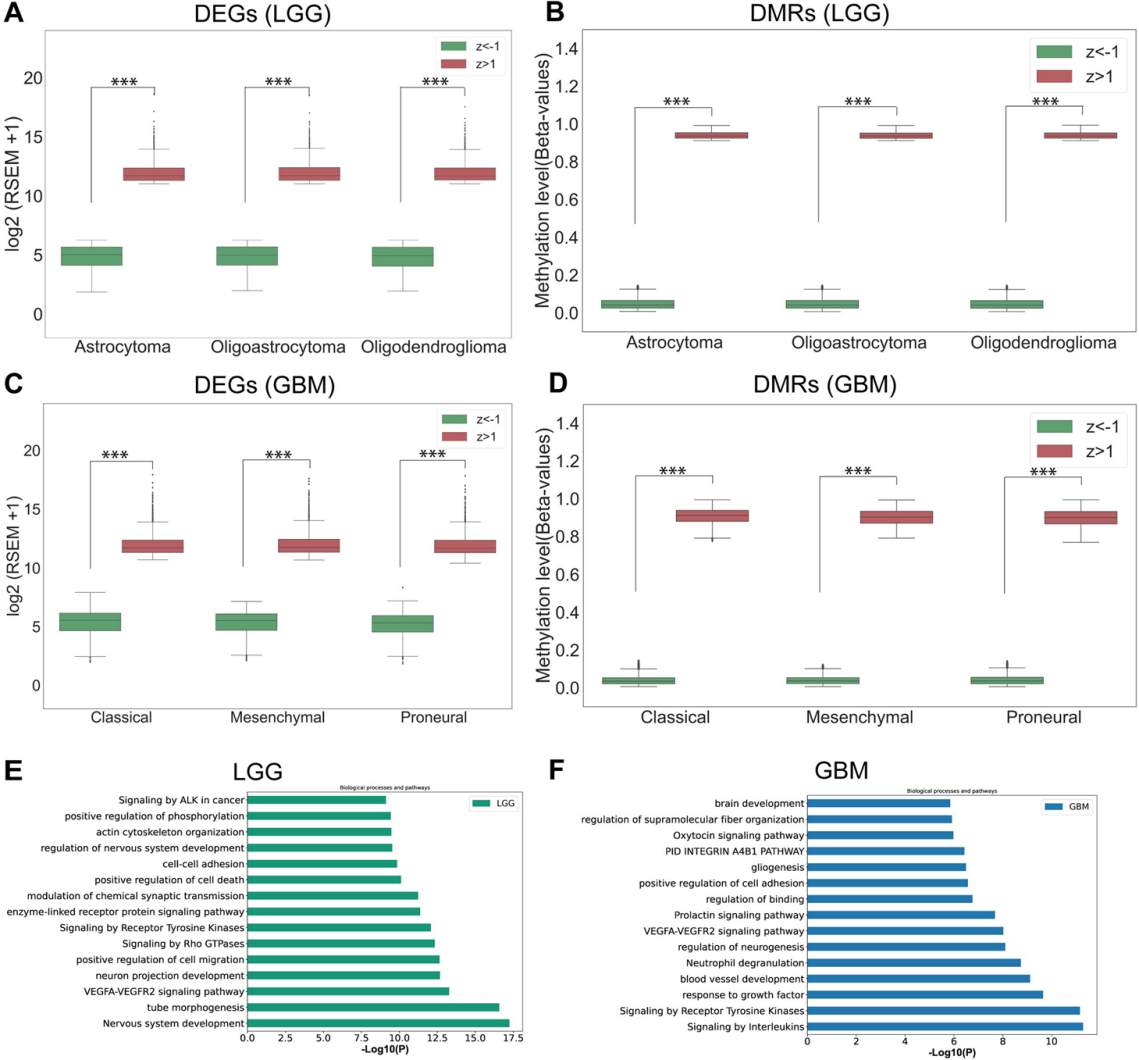
Boxplots show the difference in gene expression and methylation level between Z > 1 and Z < − 1. **A** DEGs and **B** DMRs in each LGG subtype; **C** DEGs and **D** DMRs in each GBM subtype; **E** and **F** Bar plots represent significantly enriched Biological processes and pathways of genes used as input in the autoencoder (***p < 0.001). DEGs: differentially expressed genes, DMRs: differentially methylated CpGs

Deregulated gene expression and aberrant methylation are the hallmarks of human cancer [18]. Methylation status in the promoter region determines the level of gene expression. Therefore, linking the methylome and transcriptome is crucial in finding the genetic and epigenetic features that cause cancer, which is also important for making biologically relevant models. To connect the methylome and transcriptome, patients with transcriptome and methylome profiles were chosen to identify the upregulated and downregulated genes (DEGs); and hypomethylated and hypermethylated CpGs (DMRs). We used a z-score to screen the DEGs and DMRs (see “Materials and methods” section). A z-score greater than 1 or less than − 1 indicates the gene expression and methylation are greater or less than the population mean, respectively. We identified the DEGs and DMRs for each subtype of LGG and GBM. In LGG, we found a total of 3972, 4024, and 4088 DEGs (Fig. 1A) and 177,458, 181,957, and 181,163 DMRs (Fig. 1B) in astrocytoma, oligoastrocytoma, and oligodendroglioma, respectively. In subtypes of GBM, we found a total of 3910, 3767, and 3745 DEGs (Fig. 1C), and 211,764, 208,111, and 190,743 DMRs (Fig. 1D) in classical, mesenchymal, and proneural, respectively. We also found that differences in average expression and methylation level between z > 1 and z <- 1 are statistically significant (*p*-value < 0.001) in all subtypes (Fig. 1A-D). Next, we performed a univariate Cox regression analysis to find the correlation between patient prognosis with DEGs and DMRs. We separately generated the univariate prognostic models for each DEG and DMR. Next, we screened the survival-associated genes and CpG sites based on the *p*-value < 0.05. Our results showed that, in LGG, a total of 2295 DEGs and 18,068 DMRs, and in GBM, a total of 1055 DEGs and 5033 DMRs were linked to the patient’s survival. We found that a total of 50.83% of DEGs and 20.35% of DMR in LGG; and 23.30% of DEGs and 5.41% of DMR in GBM were linked with patient survival. This indicates that a higher percentage of genes, or CpGs, are not linked with LGG or GBM. Therefore, univariate Cox analysis facilitates identifying the biologically important and cancer-associated features, which can lead to the development of a clinically relevant DL model while reducing the dimension of the data to build better-fit prediction models. Subsequently, we mapped the survival-associated CpGs in promoters (TSS1500, TSS200, the first exon, and the 5′ UTR) and their corresponding survival-associated genes. The linking of these two layers of genomic data identifies the CpG-gene pairs, which are involved in cancer progression. We found that in LGG, a total of 1110 genes (DEGs) and 3204 CpGs (DMRs) in the promoter, and in GBM, 268 genes (DEGs) and their 447 CpGs (DMRs) in the promoter are linked to patient survival (Supplementary Table 1). If a gene is involved in patient survival and if its methylation level in the promoter, which regulates its expression, is also linked to survival, this indicates an additive impact of methylation and gene expression on patient prognosis. We believe that integrating methylation levels with gene expression data will be more biologically valid for diagnostic model development. We also found that these genes (prognostic genes) are involved in biological processes and pathways that are linked to cancer (Fig. 1E and F), such as signaling by ALK in cancer [19], cell-cell adhesion [20], signaling by receptor tyrosine kinase [21], PID INTEGRIN A4B1 pathway [22], gliogenesis [23], positive regulation of cell adhesion [24] and VEGFA-VEGFR2 signaling pathways [25, 26]. Therefore, these prognostic genes and CpGs were used for autoencoder-based data integration and model building.

### Integration of gene expression and its promoter methylation level by autoencoders shows superior accuracy in subtyping

**Fig. 2:**
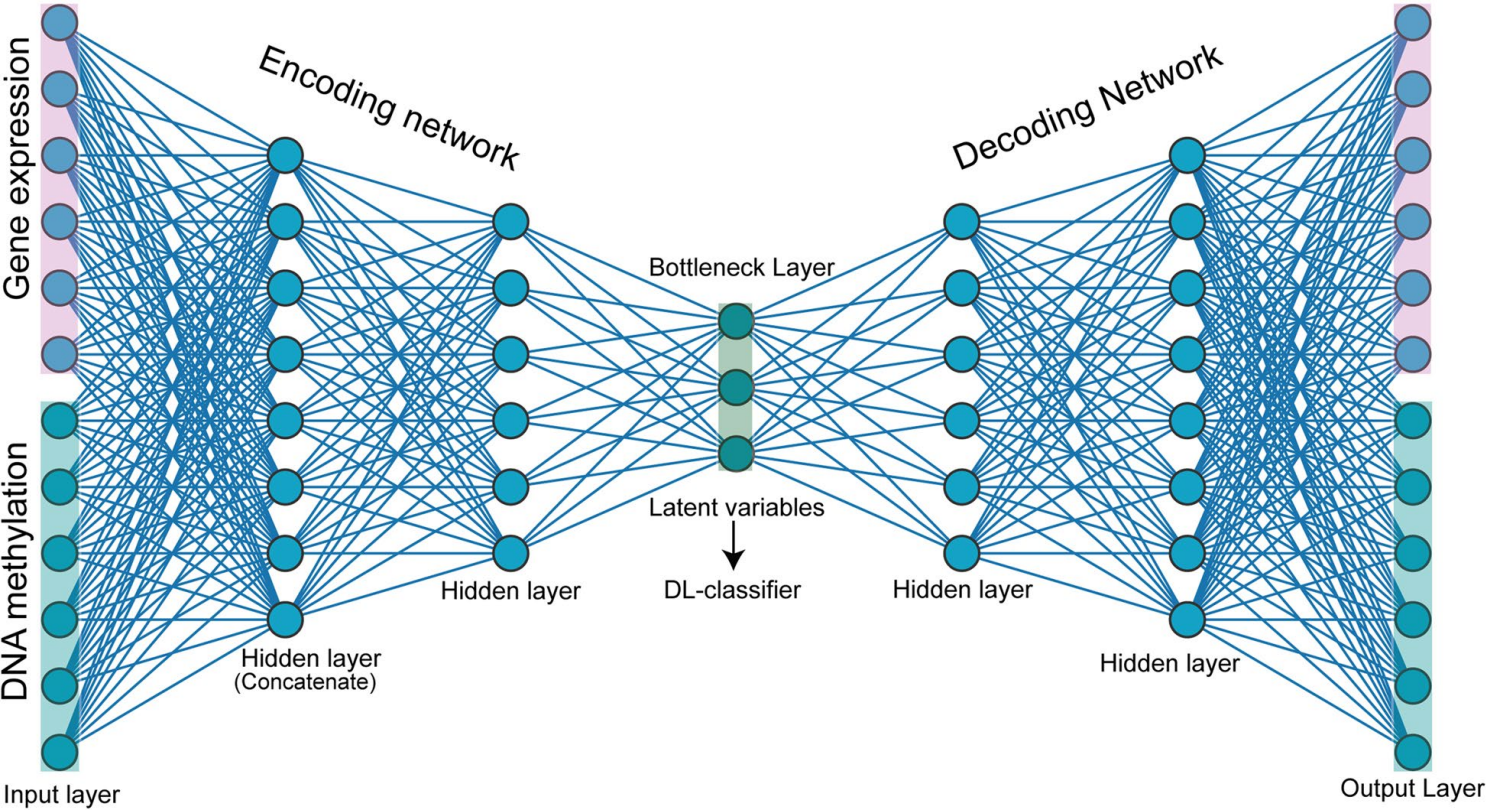
Architecture of autoencoder: The autoencoder used in DeepAutoGlioma consists of an encoder and a decoder made from 2 hidden layers and one bottleneck layer. The autoencoder has two input layers for DNA methylation and gene expression; in the first hidden layer, data is concatenated, and is passed to another hidden layer and finally compressed in the bottleneck layer. In the decoder part, the latent variables from the bottleneck layer are reconstructed to the initial ones

In the previous section, we derived the list of genes and their CpG sites in promoters linked to patient survival using univariate Cox regression analysis. Next, we extracted the gene expression and methylation matrix. We divided these datasets into training (70%) and validation (30%) sets. 70% of the data was utilized to optimize the model’s parameters and evaluate the performance of each model, and the remaining 30% of the data was employed as independent predictors. The gene expression and methylation matrices were fed into the autoencoder with concatenated inputs (CNC-AE). The methylation and gene expression levels are combined and compressed in the latent space or bottleneck layer learned by the autoencoder [14, 17, 27–29]. All the dimensions and parameters of the different layers in the autoencoder were optimized. The autoencoder consists of two parts: an encoder and a decoder network. In the encoder network, gene expression and DNA methylation profiles of LGG and GBM are first encoded into two 4314 and 715-dimensional vectors separately through hidden layers, respectively. Next, we set the dimensions of the bottleneck layers at 400 and 100 for LGG and GBM. In the decoder network, the latent variables were again used to decode the original input data, and this was used to measure the reconstruction loss, which indicates the performance of the autoencoder. The network structure of the decoder is similar to the mirror image of the encoder network (Fig. 2). If a latent variable captures the actual data pattern, i.e., intrinsic relationships between the variables, then the difference between the encoded and decoded vectors will be less. We measured the reconstruction loss using the mean squared error (MSE). We found that MSE was significantly lower, i.e., 0.04 in LGG and 0.04 in GBM. This shows that the autoencoder efficiently learned the pattern in gene expression and methylation and encoded it in the latent space. Then these latent variables were used to develop the DL models for the classification of LGG and GBM subtypes.

**Fig. 3:**
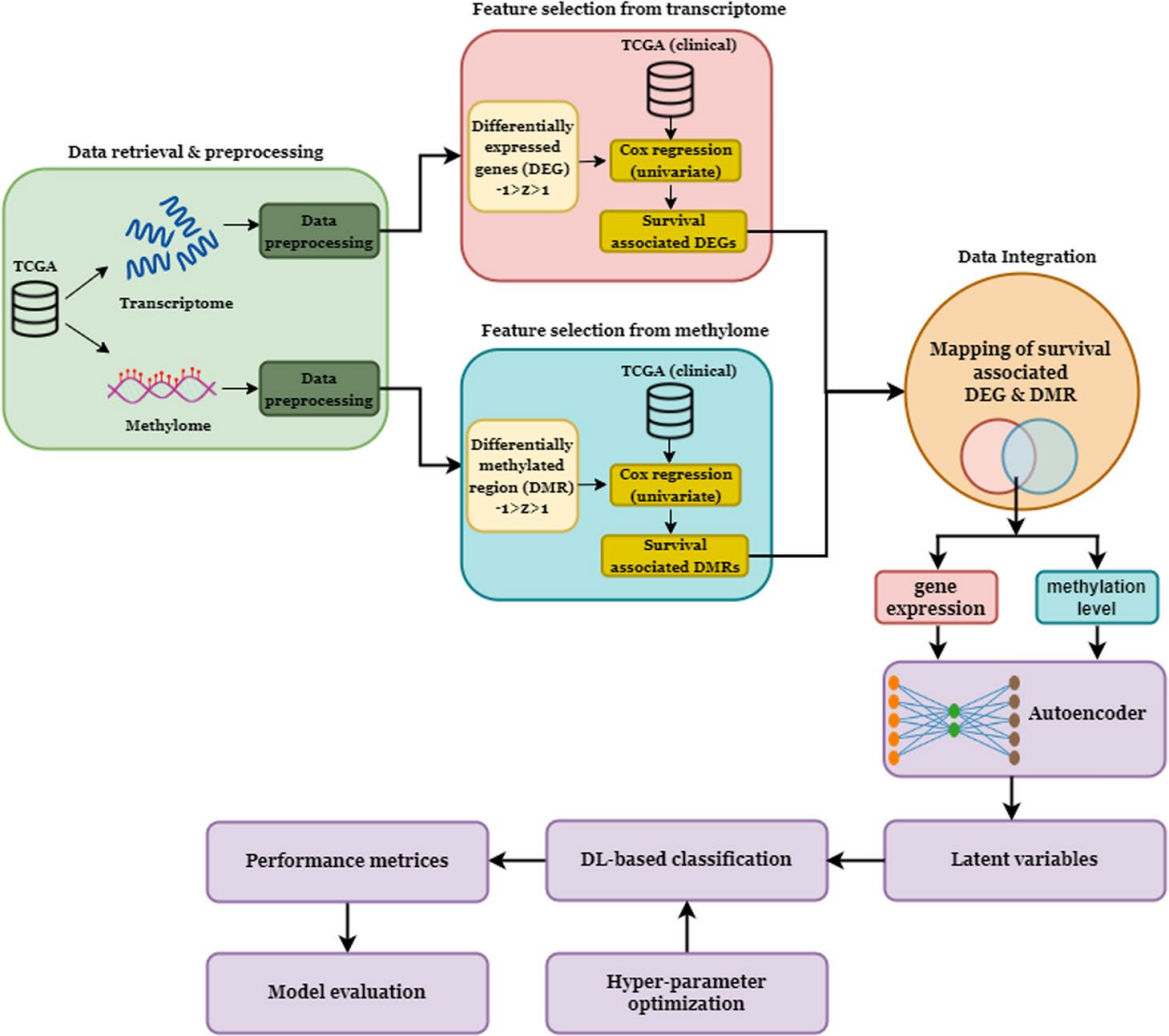
Subtype classification framework of the DeepAutoGlioma. Methylome and transcriptome data are preprocessed, differentially expressed genes (DEGs) and differentially methylated regions (DMRs) are identified, and clinically significant features are extracted. Further, these features are mapped according to the genomic region to integrate the CpG-gene pair. Then, clinically relevant methylation (CpGs) and gene expression data are fed into the autoencoder, and latent variables are extracted to build deep learning models for subtyping brain cancer

We implemented two DL algorithms, i.e., artificial neural networks (ANN) and convolutional neural networks (CNN), and compared their performance for subtype classification. During the model training step, we used the grid search method to find the best combination of hyperparameters (see material and methods). Then, using these optimal hyperparameters, we performed stratified k-fold cross-validation (k = 10) on the latent variables and computed the average performance measures for each DL model (Table 1). Average accuracy, recall, precision, F1-score, false positive rate (FPR), geometric mean (GM), and Matthew’s correlation coefficient (MCC) were used to assess the model’s performance (see materials and methods). We found that CNN models had higher prediction accuracy in subtyping, i.e., 98.03% (95% CI, 98.02–98.038) and 94.07% (95% CI, 94.04–94.10) for LGG and GBM, respectively, than the ANN models. We found that FPR, 0.01 (95% CI, 0.005–0.015), and 0.02 (95% CI, 0.01–0.03) were minimal, and the MCC scores were high,i.e., 0.96 (95% CI, 0.95–0.97) and 0.93 (95% CI, 0.89–0.97) in the case of CNN (Table 1). The higher MCC score represents a good correlation between the observed and predicted classes. Next, we performed the classification using validation datasets to check the reproducibility of the DL framework. We found the accuracy of subtype classification [for LGG 95.23% (95% CI, 95.22–95.24) and GBM 90.26% (95% CI, 90.20–90.32)] of CNN was superior, and the MCC score was 0.90 (95% CI, 0.86–0.94) and 0.92 (95% CI, 0.84–1) (Table 2). The accuracy of the current framework for subtyping LGG and GBM outperforms that of earlier machine learning (ML) and deep learning (DL) models [11, 30]. We named this framework DeepAutoGlioma (Fig. 3). We also observed the superior performance of DeepAutoGlioma using external GEO datasets (Table 3). The combination of feature genes and CpG sites in the model construction likely accounts for the impressive performance of DeepAutoGlioma. In most cases, feature selection approaches that rely on ML or DL ignore the biological relevance of features [31–33]. However, here we screened the DEGs and DMRs in each subtype, which were associated with LGG and GBM patients’ survival. Also, the genes and methylation sites used as inputs into the autoencoder are linked through their genomic locations. Together, these approaches reduce the dimension of the data, which significantly influences the model’s performance. There are very limited studies available on glioma subtype classification using multi-omics data. The study conducted by Xu et al. [34] demonstrates that the integration of multi-omics data leads to enhanced accuracy (88.50% accuracy) in the classification of glioblastoma subtypes as compared to the mono-omics data alone. However, DeepAutoGlioma exhibits a higher level of accuracy in subtype classification for both LGG and GBM. In our opinion, biologically relevant inputs to the autoencoder provided superior accuracy (95–98%) in the subtype classification achieved with CNN.

**Table 1:** Performance evaluation of LGG and GBM subtypes classification

	Methods	Performance measures (Average of 10 fold cross-validation)						
		Accuracy [95% CI]	Precision [95% CI]	Recall [95% CI]	F1-score [95% CI]	FPR [95% CI]	Gmean [95% CI]	MCC [95% CI]
**LGG**	ANN	95.40%	92.50%	92.73%	92.45%	0.03	95.28%	0.89
		[95.39–95.41]	[92.48–92.52]	[92.72–92.74]	[92.33–92.57]	[0.02–0.038]	[95.27–95.29]	[0.87–0.91]
	CNN	98.03%	97.67%	96.96%	96.97%	0.01	97.99%	0.96
		[98.02–98.038]	[97.66–97.679]	[96.95–96.97]	[96.96–96.98]	[0.005–0.015]	[97.98–97.998]	[0.95–0.97]
**GBM**	ANN	92.19%	88.05%	89.77%	87.75%	0.03	94.76%	0.9
		[92.16–92.22]	[88.00- 88.10]	[89.73–89.81]	[87.70–87.80]	[0.02–0.04]	[94.74–94.78]	[0.86–0.94]
	CNN	94.07%	90.40%	91.18%	90.25%	0.02	96.51%	0.93
		[94.04–94.10]	[90.35–90.45]	[91.13–91.23]	[90.20–90.30]	[0.01–0.03]	[96.49–96.53]	[0.89–0.97]

**Table 2:** Classification performance of deep learning algorithms on LGG and GBM subtypes for validation set

	Methods	Performance measures (Average of 10 fold cross-validation on test datset)						
		Accuracy [95% CI]	Precision [95% CI]	Recall [95% CI]	F1-score [95% CI]	FPR [95% CI]	Gmean [95% CI]	MCC [95% CI]
**LGG**	ANN	90.18%	82.40%	84.23%	82.42%	0.07	90.06%	0.8
		[98.16–98.20]	[82.35–82.45]	[84.19–84.27]	[82.37–82.47]	[0.05–0.09]	[90.04–90.08]	[0.75–0.85]
	CNN	95.23%	92.08%	92.63%	91.84%	0.03	95.30%	0.9
		[95.22–95.24]	[92.05–92.11]	[92.60- 92.66]	[91.83–91.87]	[0.02–0.04]	[95.29–95.31]	[0.86–0.94]
**GBM**	ANN	93.85%	93.85%	93.85%	93.85%	0	1	1
		[93.79–93.91]	[93.79–93.91]	[93.79–93.91]	[93.79–93.91]	[0–0]	[1–1]	[1–1]
	CNN	90.26%	85.38%	87.69%	86.15%	0.02	95.26%	0.92
		[90.20–90.32]	[85.29–85.47]	[87.62–87.76]	[86.06–86.24]	[0–0.04]	[95.21–95.31]	[0.84–1]

**Table 3:** Classification performance of DeepAutoGlioma on external datasets

	Methods	Performance measures (Average of 10 fold cross-validation)						
		Accuracy [95% CI]	Precision [95% CI]	Recall [95% CI]	F1-score [95% CI]	FPR [95% CI]	Gmean [95% CI]	MCC [95% CI]
**LGG**	ANN	91.89%	91.20%	88.00%	86.90%	0.06	92.13%	0.83
		[91.85–91.93]	[91.15–91.25]	[87.94–88.06]	[86.83–86.97]	[0.02–0.10]	[92.09–92.17]	[0.74–0.92]
	CNN	91.38%	91.38%	91.38%	91.38%	0	1	1
		[91.34–91.40]	[91.34–91.40]	[91.34–91.40]	[91.34–91.40]	[0–0]	[1–1]	[1–1]
**GBM**	ANN	84.10%	74.48%	79.48%	76.15%	0.06	90.55%	0.72
		[84.01–84.18]	[74.35–74.60]	[79.37–79.58]	[76.03–76.26]	[0.02–0.10]	[90.49–90.60]	[0.58–0.86]
	CNN	86.41%	79.87%	83.33%	81.02%	0.05	92.92%	0.76
		[86.33–86.49]	[79.75–79.99]	[83.23–83.43]	[80.90–81.13]	[0.01–0.09]	[92.87–92.97]	[0.62–0.90]

### DL models with a random feature set, preprocessed data and single omics data

**Fig. 4:**
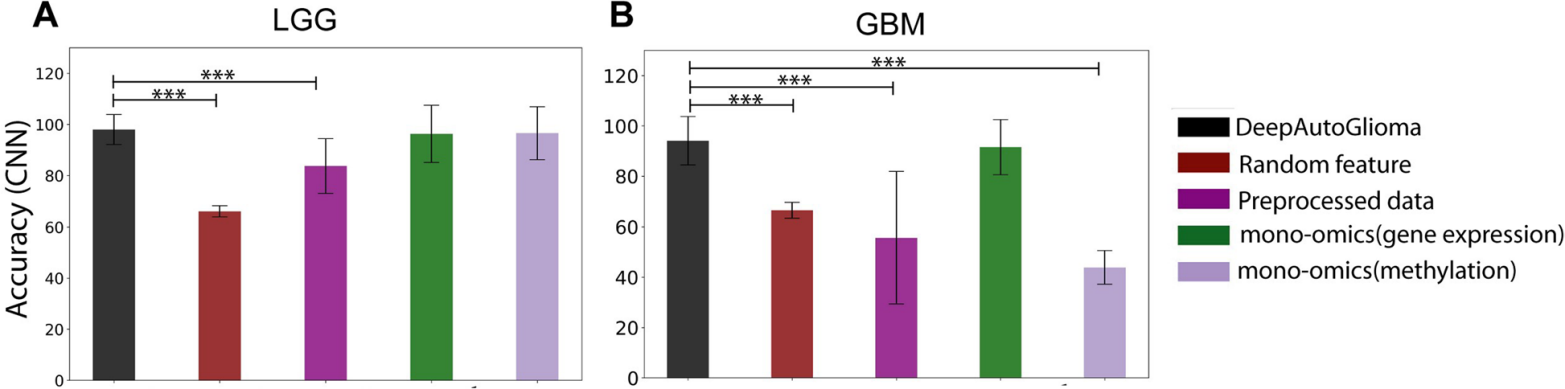
Comparison of model performance using different sets of features to that of DeepAutoGlioma (***p < 0.001)

To validate our findings and better understand the role of feature selection in model performance, we extended the DL-based model by feeding different sets of inputs (features) to the autoencoder and compared their performance to that of DeepAutoGlioma. First, we compared the performance of mapped CpGs and gene expression with randomized CpG-gene pairs as input into the autoencoder. We randomly selected the CpGs (*n* = 3204 for LGG and *n* = 447 for GBM) and genes (*n* = 1110 for LGG and *n* = 268 for GBM) from preprocessed data. Then this unmapped, randomly selected methylation and gene expression data were fed into the autoencoder. Then, ANN and CNN were used to classify the subtypes using the latent features from random datasets. We repeated the process ten times, and the accuracy varied from 60.68 to 71.43% in LGG and 62.42 to 72.14% in GBM in all iterations (Supplementary Tables 2 and 3). And the average accuracy of all iterations in CNN was 66.12 and 66.59% in LGG and GBM, respectively. When compared to DeepAutoGlioma, the average accuracy of all ten iterations in CNN is significantly less (*p*-value < 0.001, Fig. 4). Not only the accuracy, but other parameters such as precision, MCC, and FPR are very low compared to DeepAutoGlioma. This finding confirms that mapping the promoter methylation region to the gene has aided in predicting LGG and GBM subtypes with greater accuracy and precision.

To better understand the significance of biologically relevant features, such as DEGs and DMRs, as well as univariate Cox regression analysis for feature selection, we run the autoencoder on preprocessed data and then classify using ANN and CNN. LGG and GBM gene expression and methylation data matrices contain 14,517 and 14,125 genes, respectively, as well as 139,403 and 141,672 CpGs. The autoencoder was then run on these preprocessed datasets, and the accuracy of prediction, as well as other model evaluation parameters, were measured (Supplementary Table 4). When compared to DeepAutoGlioma, the prediction accuracy is significantly (*p-value* < 0.001) lower (Fig. 4). The subtype classification accuracy of LGG was 83.73% (95% CI, 83.71–83.74) in CNN and 69.86% (95% CI, 69.852–69.868) in ANN. Whereas in GBM classification, accuracy was 61.54% (95% CI, 61.53–61.55) in CNN and 67.57% (95% CI, 67.53–67.61) in ANN. Furthermore, the results of other evaluation parameters were too low to be considered. This unequivocally demonstrates that cancer-associated features or features that are biologically relevant played a crucial role in achieving higher classification accuracy.

Furthermore, we compared classification accuracy between di-omics and mono-omics data. The mono-omics data, i.e., methylation or gene expression matrix, was used as input to the autoencoder. As previously stated, we extracted compressed features from latent space, used DL algorithms, and calculated average performance metrics for each DL model. We observed that in the case of LGG, the single omics data showed good accuracy of prediction, i.e., 96.26% (95% CI, 96.25–96.27) and 96.54% (95% CI, 96.53–96.55) using gene expression and methylation data, respectively (Supplementary Table 5). However, these accuracies are lower in comparison to the DeepAutoGlioma (98.03%). However, the accuracy of prediction using test and external gene expression was 66.07% (95% CI, 66.06–66.08) and 63.81% (95% CI, 63.77–63.85), and methylation was 66.51% (95% CI, 66.50–66.52) and 74.79% (95% CI, 74.77–74.81), which is considerably less. Whereas in the case of GBM subtype prediction accuracy using gene expression and methylation data was 91.54% (95% CI, 91.53–91.57) and 43.89% (95% CI, 43.87–43.91), respectively (Supplementary Table 5). Although gene expression data showed higher accuracy, however, in the test and external datasets, the accuracy was 85.48% (95% CI, 85.37–85.59) and 72.68% (95% CI, 72.63–72.73). We saw good prediction accuracy in LGG and GBM utilizing mono-omics data, particularly gene expression, but models were unable to accurately predict subtypes using test and external datasets. This demonstrated that the individual omics data were inadequate for cancer subtype classification with superior accuracy. The models trained on multi-omics data outperformed those trained on single-omics data, owing to the fact that multi-omics data contains a wealth of information not found in a single type of omics data alone.

## Discussion

It is well established that molecular perturbations in different genomic layers cause cancer occurrence and progression. Therefore, it is crucial to perform integrative approaches that combine multi-omics data to comprehend the disease mechanism and develop novel diagnostic tools for brain cancer detection. The integration of high-throughput omics data from distinct genome layers can capture the interrelationships of biomolecules and facilitate interpreting their function in disease onset. Transcriptomics and epigenomics data are unpaired because they are usually measured in separate experiments, which demands effective and efficient in-silico multi-omics integration [28]. In the present study, we designed the deep autoencoder and deep learning (ANN and CNN)-based clinically relevant framework for integrating the methylome and transcriptome to classify the glioma subtype with superior accuracy. To strengthen the biological relevance, we screened patient samples with transcriptome and methylome profiles and measured the DEGs and DMRs in each subtype of LGG and GBM cancer. Further, we performed a univariate Cox regression analysis to identify the DEGs and DMRs associated with the patient’s survival. The univariate Cox regression approach helps to determine clinically relevant feature genes and CpG sites based on the patient’s overall survival information; further, it also decreases the data dimension. Next, we map the CpGs and genes based on the promoter regions. The linked CpGs and genes were used as input in the autoencoder. As a result, the input features in the autoencoder were biologically and clinically relevant in three ways: first, they are differentially regulated; second, they are linked to the patient’s survival; and third, methylation in the promoter is linked to gene expression. We found that using latent variables learned by the autoencoder as an input in deep learning models (ANN and CNN), we were able to predict the subtypes of LGG and GBM with an accuracy of 98.03% (95% CI, 98.02–98.038) and 94.07% (95% CI, 94.04–94.10), respectively, using CNN. Furthermore, the current framework classifies the GBM and LGG subtypes using the external datasets with 86.41% (95% CI, 86.33–86.49) and 91.89% (95% CI, 91.85–91.93) accuracy, respectively. On the other hand, autoencoder-based deep learning with omics data, randomized CpG-gene pair, and preprocessed dataset did not perform well compared to DeepAutoGlioma. We believe that feature screening using various statistical methods and integration of di-omics data using autoencoders play an essential role in achieving higher subtyping accuracy. The current study demonstrated how data integration could lead to the discovery of novel patterns in transcriptomics and epigenomics data and aid in developing efficient diagnostic tools. It is anticipated that multimodal learning approaches for multi-omics data analysis will become more prevalent in cancer diagnosis, allowing physicians to more accurately determine the most effective line of therapy. We hope the current DL framework will assist clinicians in personalizing treatment for brain cancer patients, which could lead to better treatment outcomes.

## Conclusions

The accurate subtyping of gliomas is crucial for precision therapy. A DL-based model can improve the overall precision and efficacy of diagnostic processes using large-scale omics data. However, clinical diagnosis still raises questions about the validity and interpretability of DL- or AI-based diagnostic models. Therefore, it is essential to design a biologically and clinically relevant AI-based diagnostic model to increase the reliability of diagnosis. Here we design the AI-based diagnostic tool, i.e., DeepAutoGlioma, for subtyping the glioma. The transcriptome and methylome data of glioma patients were used to extract biologically and clinically relevant features for model development. The features from two levels of genomic layers were integrated to capture cancer-specific patterns for accurate subtyping. Integration of omics data enables us to achieve greater model performance because it provides a wealth of information from different genomic layers. The model developed based on multi-omics data can greatly support the clinician in personalizing treatment.

## Materials and methods

### Data collection and preprocessing

We retrieved methylome (Illumina Infinium HumanMethylation450 platform) and transcriptome (RNA-seq) data of TCGA from UCSC Xena (https://xena.ucsc.edu) [35] log2 (RSEM + 1) values for gene expression and beta-values for methylation levels were considered for analysis. Here, RSEM stands for RNA-Seq by Expectation Maximization. Next, low-expressed genes were filtered out of the transcriptome data [log2 (RSEM + 1) < 0.1 in the 90% sample]. Patients with both transcriptome and methylome profiles were considered for analysis. GBM patients (*n* = 52) were divided into three groups based on their clinical information: classical (*n* = 16), mesenchymal (*n* = 22), and proneural (*n* = 14). Similarly, the LGG patients (*n* = 281) were divided into three groups based on cancer subtype, i.e., astrocytoma (*n* = 96), oligoastrocytoma (*n* = 75), and oligodendroglioma (*n* = 110). We obtained the external data set from the Gene Expression Omnibus (GEO) repository. The subtyping of LGG was validated using GSE74462, GSE43378 (gene expression data), and GSE129477 (DNA methylation data). The subtyping of GBM was validated using the gene expression data from GSE145645 and the DNA methylation data from GSE128654.

### Identification of differentially expressed genes and differentially methylated regions

DEGs and DMRs were identified by z-score. We classified high- and low-expressed genes as well as hyper- and hypo-methylated CpG sites using the Z-score because healthy patient data for the transcriptome and methylome were unavailable. The following formula was used to determine the Z-score for each gene or CpG site in a certain subtype:

$$Z-score=\frac{\overset-x-\mu}\sigma$$

Here, $\underset{\_}{x}$denotes the subtype-specific average gene expression or methylation level, whereas $\mu$ and $\sigma$ stand for the population mean and population standard deviation, respectively. For each subtype of LGG and GBM, we used Z-score > 1 for higher expression and hypermethylation and Z-score < -1 for lower expression and hypomethylation. Then, considering that differential methylation in the promoter regions may affect the related gene’s expression, we screened the higher- and lower-expressed genes whose promoter regions were differentially methylated. Finally, genes with differential expression and methylated promoter regions were used for further analysis.

### Construction of univariate cox regression models and survival analysis

Univariate Cox regression analysis was implemented to build the prognostic risk-score model for a particular gene and CpG site [36]. Univariate Cox regression analysis was performed using the survminer and survival package in R. The *p*-value < 0.05 was considered the significant association of a gene or CpG site with patients’ overall survival (OS).

$$h(t)=h_0(t)\times exp\left\{b_1x_1+b_2x_2+........+b_px_p\right\}$$

Where t is survival time, h(t) is the hazard function determined by a set of covariates (${x}_{1}$, ${x}_{2}$, *…….*, ${x}_{p}$) for genes or methylation sites,${b}_{1}$, ${b}_{2}$, *…….*, ${b}_{p}$ are the coefficients of regression, ${h}_{1}$ is a baseline hazard.

### Mapping and integration of methylation and gene expression data

CpG IDs and genes were mapped through the promoter region. The TSS1500, TSS200, the first exon, and the 5′ UTR were considered promoters of a gene. If both gene expression and methylation levels at the promoter alter (i.e., DEGs and DMRs), then we screened those CpG-gene pairs. Next, we constructed methylation and gene expression matrices using these CpGs and genes fed in an autoencoder using two separate layers.

### Biological processes and pathway enrichment analysis

We analyzed biological processes and pathway enrichment using the Metascape tool [37]. Enrichment analysis was performed using the following ontology sources: Gene Ontology (GO) Biological Processes, KEGG Pathway and Reactome Gene Sets, and the Kyoto Encyclopedia of Genes and Genomes (KEGG). If the adjusted *p*-value < 0.05, the biological process or pathway was considered significantly enriched.

### Autoencoder implementation

Autoencoders are feed-forward neural networks that aim to copy the input variable to the output variable with the minimum loss of information. It compresses the inputs into latent variables in the bottleneck layer’s embedding space and then reconstructs the output from the embedding space. The autoencoder is composed of two parts: the encoder and the decoder. The encoder maps the high dimensional input data into latent variables in embedding space, and the decoder reconstructs the input data from the embedding. Here, we used one concatenated layer, one hidden layer, and a bottleneck layer in the encoding part. We used a concatenated autoencoder to integrate the gene expression and methylation data. We used the Keras library with TensorFlow [38] to implement the concatenated autoencoder. To integrate the gene expression and methylation level of LGG in the hidden layer of the autoencoder, a rectified linear activation function (ReLU) was used. In the bottleneck layer, a uniform kernel initializer and a linear activation function were implemented. Similarly, in the decoding layer, one hidden layer and a concatenated layer were also used. ReLU activation function was applied to the decoder layer. The same architecture we have followed in the GBM dataset for integrating the gene expression and methylation data. In the GBM dataset, the Exponential Linear Unit (ELU) activation function was used in the hidden layers. Linear activation function and a uniform kernel initializer were employed at the bottleneck layer. Further, the ELU activation function is applied to the decoder layer. Epoch size and batch size were 1500 and 16, respectively, in each dataset. The architecture of the autoencoder is shown in Fig. 2. We selected 1110 features from gene expression and 3204 features from DNA methylation in the LGG dataset, while in the GBM dataset, 268 features from gene expression, 447 features from DNA methylation were selected for the input layer. For the autoencoder, we set a concatenate layer, hidden layers, and a bottleneck layer, respectively. We obtained the 400 and 100 features from the bottleneck in the LGG and GBM datasets, respectively.

### Deep learning classifier

ANNs, which imitate the human brain, are feed-forward neural networks. ANNs are represented by a weighted, directed graph connecting inputs to a series of interconnected “hidden” layers that are composed of multiple nodes called “neurons” that are in turn connected to an output layer [39]. ANNs are trained to recognize and categorize complex patterns. There is one input layer, one output layer, and one hidden layer in the network. The hidden layers lie between the input and output layers. The number of output neurons varies depending on the specific application, while the number of input neurons is equal to the number of attributes. Here, latent variables obtained from the bottleneck layer of the autoencoder were used as input. The parameters were optimized on training datasets with the grid search method using the GridSearchCV package in Python used for classifying the LGG and GBM by implementing ANN method: for LGG the parameters are, activation = relu, batch size = 32, epochs = 100, and optimizer = adam; for GBM, the parameters are activation = linear, batch size = 30, epochs = 50, and optimizer = RMSprop. We have used Keras library to build ANN deep-learning classifiers on the Python platform.

CNN is a type of deep learning method that directly learns from the data. CNN consists of three layers: convolutional, pooling and fully connected (FC) layers [40]. The convolutional layer is the first layer, while the FC layer is the last. In the first layer, i.e., the convolutional layer, where filters are applied to raw data or feature maps in deep CNN, convolution is one linear operation utilized in place of generic matrix multiplication. The convolution operation (denoted by an asterisk) is defined by:

$$f\left(t\right)=\left(x*K\right)\left(t\right)$$

where the function $x \left(t\right)$ is referred to as input, $K\left(t\right)$ is referred to as kernel, and the $f\left(t\right)$ is referred to as output. After the convolutional layer, the input data is downscaled by the pooling layer to save computation, and the final prediction is made by the fully connected layer. Since every node in a single layer is fully connected to every node in the subsequent layer, it represents a network that is fully connected. Keras library was used to construct the model architecture for CNN. Eight convolutional layers were used to obtain the best result. Furthermore, parameters were optimized with the grid search method using the GridSearchCV package in Python. The parameters were used for classifying the LGG and GBM by implementing CNN are: for LGG the parameter are, activation = relu, batch size = 64, dropout rate = 0.2, epochs = 2000, filters = 1, kernel size = 3, optimizer = RMSprop; for GBM the parameters are, activation = elu, batch size = 64, dropout rate = 0.2, epochs = 2000, filters = 1, kernel size = 3, optimizer = RMSprop. Using a stratified k-fold CV, the 70% training dataset was used to calculate the average performance of the model using the optimal parameters. In a stratified k-fold CV, the dataset is split into k different folds, of which k-1 was utilized to train the network, and the final fold was set aside for testing. This procedure is then repeated until all folds are used once as a test set. The final output is then computed by averaging the performance parameters obtained from each test set.

### Performance evaluation

The performance of the DL model was evaluated based on the eight criteria: accuracy, sensitivity, specificity, precision, F1-score, FPR, geometric mean, and MCC. A true positive (TP) would indicate that the cancerous cell is identified correctly, while a false positive (FP) indicates that the cancerous cell is identified as healthy. Conversely, true negatives (TN) and false negatives (FN) are calculated for healthy cells. The following equations define the metrics:

$$Accuracy=\frac{TP+TN}{TP+TN+FP+FN}$$

$$Sensitivity=\frac{TP}{TP+FN}$$

$$Specificity=\frac{TN}{TN+FP}$$

$$Precision=\frac{TP}{TP+FP}$$

$$F1-score=\frac{2\times TP}{2\times TP+FP+FN}$$

$$FPR=\frac{FP}{TN+FP}$$

$$Geometric\;mean=\sqrt[n]{(x_1.x_2\dots\dots.x_n)}$$

$$MCC=\frac{\left(TP\times TN\right)- (FP\times FN)}{\sqrt{\left(TP+FP\right)\left(TP+FN\right)\left(TN+FP\right)(TN+FN)}}$$

We used the sklearn.metrics library in Python to calculate the above score by importing functions such as confusion_matrix and classification_performance.

### Statistical analysis

Pairwise comparison was done using the Mann-Whitney U test using Sigma Plot 11.0.

### Supplementary Information

**Additional file 1: Supplementary Table 1. **List of input features in autoencoder.

**Additional file 2: Supplementary Table 2.** Model performance in LGG subtype classification using random features.

**Additional file 3: Supplementary Table 3.** Model performance in GBM subtype classification using random features.

**Additional file 4: Supplementary Table 4.** Model performance in LGG and GBM subtyping using preprocessed data as a feature.

**Additional file 5: Supplementary Table 5.** Classification performance of deep learning algorithms on LGG and GBM subtyping using mono-omics data.

## Data Availability

Cancer patient transcriptome, methylome, and clinical data of LGG and GBM is available in UCSC Xena database (https://xenabrowser.net/datapages/). The external datasets were retrieved from GEO (https://www.ncbi.nlm.nih.gov/geo/).
