# Supplementary material for: DeepAutoGlioma: a deep learning autoencoder-based multi-omics data integration and classification tools for glioma subtyping
Source: BioData Min. 2023 Nov 15;16:32. doi: 10.1186/s13040-023-00349-7 (PMC10652591; doi:10.1186/s13040-023-00349-7)
Supplement: Supplementary file 2 — Additional file 2: Supplementary Table 2. Model performance in LGG subtype classification using random features. [file 13040_2023_349_MOESM2_ESM.docx]

**Supplementary Table 2** Model performance in LGG subtype classification using random features

|  | **Methods** | **Performance measures (Average of 10 fold cross-validation)** | | | | | | |
| --- | --- | --- | --- | --- | --- | --- | --- | --- |
|  |  | **Accuracy** | **Precision** | **Recall** | **F1-score** | **FPR** | **Gmean** | **MCC** |
| **Iteration1** | ANN | 64.02%(±0.08) | 45.77 | 43.67 | 43.51 | 0.26 | 64.22 | 0.2 |
|  | CNN | 64.09%(±0.05) | 43.51 | 43.43 | 42.5 | 0.26 | 64.24 | 0.2 |
| **Iteration2** | ANN | 60.68%(±0.04) | 23.49 | 33.52 | 22.72 | 0.28 | 58.33 | 0.08 |
|  | CNN | 62.44%(±0.08) | 31.62 | 37.27 | 28.9 | 0.27 | 60.23 | 0.11 |
| **Iteration3** | ANN | 69.06%(±0.08) | 53.01 | 51.34 | 51.55 | 0.22 | 68.77 | 0.3 |
|  | CNN | 70.62%(±0.08) | 55.78 | 53.29 | 52.82 | 0.22 | 70.34 | 0.33 |
| **Iteration4** | ANN | 68.79%(±0.09) | 51.89 | 51.21 | 51.05 | 0.23 | 68.92 | 0.3 |
|  | CNN | 67.29%(±0.07) | 47.83 | 48.09 | 47.03 | 0.24 | 67.52 | 0.27 |
| **Iteration5** | ANN | 65.69%(±0.10) | 47.81 | 47.81 | 46.48 | 0.25 | 65.69 | 0.23 |
|  | CNN | 68.24%(±0.07) | 50.68 | 51.05 | 49.36 | 0.24 | 68.33 | 0.29 |
| **Iteration6** | ANN | 64.65%(±0.07) | 45.16 | 44.47 | 43.76 | 0.26 | 64.35 | 0.2 |
|  | CNN | 64.23%(±0.06) | 44.92 | 44.12 | 43.53 | 0.26 | 63.75 | 0.18 |
| **Iteration7** | ANN | 69.14%(±0.04) | 53.62 | 51.78 | 50.93 | 0.23 | 69.08 | 0.31 |
|  | CNN | 65.96%(±0.07) | 47.14 | 45.73 | 44.67 | 0.25 | 65.74 | 0.23 |
| **Iteration8** | ANN | 68.21%(±0.05) | 51.45 | 49.82 | 48.8 | 0.23 | 68.22 | 0.28 |
|  | CNN | 65.72%(±0.06) | 47.76 | 47.07 | 45.88 | 0.25 | 65.75 | 0.24 |
| **Iteration9** | ANN | 64.9%(±0.11) | 45.71 | 44.66 | 43.87 | 0.26 | 64.9 | 0.21 |
|  | CNN | 65.74%(±0.08) | 46.01 | 46.24 | 44.58 | 0.25 | 65.82 | 0.24 |
| **Iteration10** | ANN | 71.43%(±0.06) | 55.35 | 54.88 | 53.7 | 0.21 | 71.56 | 0.36 |
|  | CNN | 66.88%(±0.05) | 50.12 | 48.96 | 48.47 | 0.24 | 66.88 | 0.26 |
