# Supplementary material for: DeepAutoGlioma: a deep learning autoencoder-based multi-omics data integration and classification tools for glioma subtyping
Source: BioData Min. 2023 Nov 15;16:32. doi: 10.1186/s13040-023-00349-7 (PMC10652591; doi:10.1186/s13040-023-00349-7)
Supplement: Supplementary file 3 — Additional file 3: Supplementary Table 3. Model performance in GBM subtype classification using random features. [file 13040_2023_349_MOESM3_ESM.docx]

**Supplementary Table 3** Model performance in GBM subtype classification using random features

|  | **Methods** | **Performance measures (Average of 10 fold cross-validation)** | | | | | | |
| --- | --- | --- | --- | --- | --- | --- | --- | --- |
|  |  | **Accuracy** | **Precision** | **Recall** | **F1-score** | **FPR** | **Gmean** | **MCC** |
| **Iteration1** | ANN | 64.59%(±0.11) | 31.46 | 43.83 | 35.25 | 0.24 | 64.40 | 0.25 |
|  | CNN | 62.42%(±0.16) | 39.26 | 43.68 | 38.79 | 0.27 | 60.67 | 0.17 |
| **Iteration2** | ANN | 69.55%(±0.16) | 46.55 | 57.47 | 47.91 | 0.22 | 68.81 | 0.39 |
|  | CNN | 71.49%(±0.12) | 50.88 | 56.94 | 49.59 | 0.21 | 70.36 | 0.38 |
| **Iteration3** | ANN | 69.68%(±0.18) | 53.81 | 60.21 | 52.84 | 0.22 | 68.71 | 0.39 |
|  | CNN | 70.31%(±0.16) | 61.30 | 62.06 | 57.24 | 0.21 | 68.56 | 0.38 |
| **Iteration4** | ANN | 66.59%(±0.18) | 46.90 | 48.83 | 45.35 | 0.26 | 61.95 | 0.27 |
|  | CNN | 62.64%(±0.18) | 35.44 | 43.66 | 36.27 | 0.28 | 61.94 | 0.18 |
| **Iteration5** | ANN | 65.64%(±0.12) | 39.77 | 46.62 | 40.61 | 0.25 | 64.85 | 0.25 |
|  | CNN | 69.71%(±0.13) | 51.31 | 57.76 | 50.84 | 0.22 | 69.20 | 0.38 |
| **Iteration6** | ANN | 65.09%(±0.12) | 38.56 | 48.02 | 39.36 | 0.26 | 64.46 | 0.25 |
|  | CNN | 65.01%(±0.14) | 33.05 | 48.04 | 37.55 | 0.26 | 63.70 | 0.24 |
| **Iteration7** | ANN | 68.64%(±0.17) | 43.20 | 51.90 | 44.94 | 0.24 | 67.29 | 0.29 |
|  | CNN | 68.47%(±0.07) | 46.11 | 56.26 | 47.58 | 0.22 | 67.63 | 0.37 |
| **Iteration8** | ANN | 68.13%(±0.11) | 37.23 | 45.75 | 39.30 | 0.22 | 65.76 | 0.26 |
|  | CNN | 63.19%(±0.15) | 32.73 | 41.66 | 34.25 | 0.26 | 62.49 | 0.23 |
| **Iteration9** | ANN | 72.14%(±0.17) | 51.47 | 53.64 | 50.68 | 0.20 | 71.29 | 0.40 |
|  | CNN | 67.36%(±0.08) | 42.73 | 52.43 | 44.16 | 0.24 | 66.53 | 0.32 |
| **Iteration10** | ANN | 65.19%(±0.10) | 33.65 | 48.04 | 37.07 | 0.24 | 64.09 | 0.26 |
|  | CNN | 65.36%(±0.15) | 39.78 | 50.04 | 41.71 | 0.26 | 63.02 | 0.26 |
