# Supplementary material for: DeepAutoGlioma: a deep learning autoencoder-based multi-omics data integration and classification tools for glioma subtyping
Source: BioData Min. 2023 Nov 15;16:32. doi: 10.1186/s13040-023-00349-7 (PMC10652591; doi:10.1186/s13040-023-00349-7)
Supplement: Supplementary file 4 — Additional file 4: Supplementary Table 4. Model performance in LGG and GBM subtyping using preprocessed data as a feature. [file 13040_2023_349_MOESM4_ESM.docx]

**Supplementary Table 4** Model performance in LGG and GBM subtyping using preprocessed data as a feature

|  | **Methods** | **Performance measures (Average of 10 fold cross-validation)** | | | | | | |
| --- | --- | --- | --- | --- | --- | --- | --- | --- |
|  |  | **Accuracy [95% CI]** | **Precision [95% CI]** | **Recall [95% CI]** | **F1-score [95% CI]** | **FPR [95% CI]** | **Gmean [95% CI]** | **MCC [95% CI]** |
| **LGG** | ANN | 69.86% | 53.45% | 53.39% | 50.46% | 0.22 | 70.22% | 0.34 |
|  |  | [69.852 - 69.868] | [53.44 - 53.46] | [53.37 - 53.402] | [50.448 - 40.472] | [0.215 - 0.225] | [70.216 - 70.224] | [0.321 - 0.359] |
|  | CNN | 83.73% | 75.77% | 73.99% | 73.40% | 11.00% | 83.50% | 0.63 |
|  |  | [83.71 - 83.74] | [75.75 - 78.78] | 73.97 - 74.01] | 73.38 - 73.42] | [0.01 - 0.119] | [83.488 - 83.512] | [0.61 - 0.65] |
| **GBM** | ANN | 67.57% | 39.23% | 48.83% | 41.05% | 0.24 | 65.89% | 0.26 |
|  |  | [67.53 - 67.61] | [39.20 - 39.30] | [48.77 - 48.89] | [40.98 - 41.10] | [0.22 - 0.26] | [65.85 - 65.93] | [0.16 - 0.36] |
|  | CNN | 61.54% | 34.76% | 44.05% | 36.26% | 29.00% | 61.35% | 0.17 |
|  |  | [61.53 - 61.55] | [34.68 - 34.84] | [43.98 - 44.12] | [36.19 - 36.33] | [0.26 - 0.32] | [61.34 - 61.36] | [0.06 - 0.28] |
