# Supplementary material for: DeepAutoGlioma: a deep learning autoencoder-based multi-omics data integration and classification tools for glioma subtyping
Source: BioData Min. 2023 Nov 15;16:32. doi: 10.1186/s13040-023-00349-7 (PMC10652591; doi:10.1186/s13040-023-00349-7)
Supplement: Supplementary file 5 — Additional file 5: Supplementary Table 5. Classification performance of deep learning algorithms on LGG and GBM subtyping using mono-omics data. [file 13040_2023_349_MOESM5_ESM.docx]

**Supplementary Table 5** Classification performance of deep learning algorithms on LGG and GBM subtyping using mono-omics data

|  |  | **Methods** | **Performance measures (Average of 10 fold cross-validation)** | | | | | | |
| --- | --- | --- | --- | --- | --- | --- | --- | --- | --- |
|  |  |  | **Accuracy [95% CI]** | **Precision [95% CI]** | **Recall [95% CI]** | **F1-score [95% CI]** | **FPR [95% CI]** | **Gmean [95% CI]** | **MCC [95% CI]** |
| **LGG** | **only Gene expression** | ANN | 94.70% | 91.21% | 91.40% | 91.29% | 0.03 | 94.63% | 0.87 |
|  |  |  | [94.69 - 94.71] | [91.19 - 91.23] | 91.39 - 91.43] | [91.27 - 91.31] | [0.02 - 0.04] | [94.62 - 94.64] | [0.84 - 0.90] |
|  |  | CNN | 96.26% | 94.24% | 94.29% | 94.15% | 0.02 | 96.30% | 0.91 |
|  |  |  | [96.25 - 96.27] | [94.22 - 94.26] | [94.27 - 94.31] | [94.13 - 94.17] | [0.01 - 0.03] | [96.29 - 96.31] | [0.88 - 0.94] |
|  | **only DNA Methylation** | ANN | 92.61% | 88.21% | 87.72% | 87.72% | 0.05 | 92.51% | 0.83 |
|  |  |  | [92.60 - 92.62] | [88.23 - 88.27] | [87.69 - 87.75] | [87.69 - 87.75] | [0.04 - 0.06] | [92.50 - 92.52] | [0.79 - 0.87] |
|  |  | CNN | 96.54% | 93.81% | 94.31% | 94.03% | 0.02 | 96.60% | 0.92 |
|  |  |  | [96.53 - 96.55] | [93.79 - 93.83] | [94.29 - 94.33] | [94.01 - 94.05] | [0.01 - 0.03] | [96.59 - 96.61] | [0.89 - 0.95] |
| **GBM** | **only Gene expression** | ANN | 85.91% | 73.87% | 78.83% | 74.24% | 0.08 | 88.90% | 0.8 |
|  |  |  | [85.88 - 85.94] | [73.80 - 73.94] | [73.77 - 73.89] | [74.17 - 74.31] | [0.06 - 0.10] | [88.88 - 88.92] | [0.75 - 0.80] |
|  |  |  | 91.54% | 85.24% | 88.45% | 85.90% | 0.04 | 94.14% | 0.89 |
|  |  | CNN | [91.53 - 91.57] | [85.17 - 85.31] | [88.40 - 88.50] | [85.84 - 85.96] | [0.02 - 0.06] | [94.12 - 94.16 | [0.84 - 0.94] |
|  | **only DNA Methylation** | ANN | 44.59% | 42 67% | 11.87% | 18.52% | 0.2 | 43.13% | 0 |
|  |  |  | [44.58 - 44.60] | [42.63 - 42.71] | [11.86 - 11.88] | [18.50 - 18.54] | [0.194 - 0.206] | [43.09 - 43.17] | [0 - 0] |
|  |  | CNN | 43.89% | 42.67% | 13.05% | 19.77% | 0.19 | 44.88% | 0 |
|  |  |  | [43.87 - 43.91] | [42.63 - 42.71] | [13.04 - 13.06] | [19.75 - 19.79] | [0.181 - 0.199] | [44.86 - 44.90] | [0 - 0] |
